# John Howard's Observations on Hospitals (1773-1790)

**Published:** 1905-11-25

**Authors:** Henry M. Hurd

**Affiliations:** Superintendent of the Johns Hopkins Hospital, Baltimore, Md.


### HOSPITAL ADMINISTRATION.

### CONSTRUCTION AND ECONOMICS.

### JOHN HOWARD'S OBSERVATIONS ON HOSPITALS (1773-1790).

By Henry M. Hurd, M.D., Superintendent of the Johns Hopkins Hospital, Baltimore, Md.

(*Continued from page* 121.)

Howard on Construction and Organisation.

These observations upon infirmaries in connection with prisons thus prepared his mind to deal with the more complex problems of hospital construction and organisation. As illustrative of the character of his mind, and his method of inspecting hospitals, I have copied a few of his notes, taken almost at random, from the two books to which I have referred. Thus, after an inspection of the institutions at Amsterdam, he writes:

I visited the pest-house and several hospitals for the sick, but shall only observe in general the impropriety of keeping the patients too warm by placing their beds with woollen curtains in small recesses or cupboards in the walls. The women's ward in the gast-huys, which was formerly a church, is an exception. This is lofty and spacious with windows opposite to one another and a stone floor; and though full of patients it was not in the least offensive.

In the orphan-house at Amsterdam there were about 1,300 children of both sexes. The rooms for the directors and the kitchen were neat and clean; but the bedrooms were close and unhealthy, being crowded with beds with three or four children for each bed. The infirmaries were situated on the ground floor; and the beds in them with two or three children in each enclosed in boxes in the walls. For want of air, the workrooms, schoolrooms, and refectories were so unhealthy and offensive that the children here (as in three other orphan-houses in this city and in the orphan-house at Rotterdam) are indeed objects of great compassion. Many of the servants in these houses are old and indolent; the children miserably nasty and most of them troubled with scorbutic and cutaneous disorders to a great degree. On observing this to some of the directors they replied in words that gave me pain and excited my indignation, "It is the house disorder; all our children must have a seasoning." Thus do the physicians and governors excuse the abuse of their trust. The consequence must be that few of the children reach manhood and that such as do are a feeble and sickly race.

After visiting the Catherine and Paulowski hospistals at Moscow, he writes: "The foundation of this and Paulowski being stone, and elevated considerably above the level of the ground, I observed several apertures (12 in. by 7) in the stone-work for causing a circulation of air under the floors. If the apertures were also made in the floors, they would be conducive to health by freshening and airing the rooms." In Warsaw he found the following: "In the Hospital of St. Lazarus, appropriated to venereal patients, there were 61 miserable objects in close, offensive rooms. It is badly situated, and in all respects one of the worst hospitals I ever saw."

Speaking of a hospital at Civita Vecchia, he says: "A particular room was appropriated for such as had cutaneous disorders, and another for consumptive patients. In this country the physicians are persuaded that the consumption is a contagious disorder. Patients afflicted with it in hospitals have a separate ward. The same precautions are used to prevent infection as in the plague. When this disorder has been in private houses the furniture is destroyed and the rooms are scraped and fumigated before they are inhabited." Again, while in Madrid, he writes: "Here are likewise rooms (carefully separated) for insane, for dropsical, and for consumptive patients. The contagion of consumption is supposed to infect not only the clothes, bedding, and furniture of rooms, but also the walls and ceiling. Danger has been apprehended even from the horses of consumptive patients; and for this reason it was thought necessary in an instance which was mentioned to me to kill the horse of an officer who had died of this distemper."

Defects in London Hospitals in Howard's Day.

After giving an excellent account of all the hospitals in London, he makes the following remarks:

I shall beg leave to subjoin a few general observations concerning defects in the London hospitals, premising that I fear the public attention to them is much relaxed of late years in consequence of the newer establishments of dispensaries which have multiplied so as to injure the funds of the older institutions. The securities and fees required at admission into many of the hospitals bear hard upon the poor and absolutely exclude many of those who have the greatest occasion for charitable relief. The nurses' fees in particular open a door to many impositions. The visits of Governors are too often only a matter of form, the visitor hurrying out of an offensive room, and readily acquiescing in the reports of nurses, etc. Hence I apprehend many instances of neglect in surgeons and their dressers as well as other officers go unnoticed. I have never found any clergyman administering consolation and admonition to the sick; and prayers are usually attended by very few. Whitewashing the wards is seldom or never practised, and injurious prejudices against washing floors and admitting fresh air are suffered to operate. Bathing, hot or cold, is scarcely ever used; I suppose because it would give trouble to the attendants. There are no convalescent or sitting-rooms, so that patients are often turned out very unfit for work or the common mode of living. The admission of great quantities of beer for patients from ale-houses by alleged or pretended orders from the faculty is a great and growing evil. Every proper article of diet should be provided by the hospital and no other on any account be admitted. It is a pity that for want of attention to these circumstances, such noble institutions should be rendered of much less public utility than was intended by their governors, founders, and supporters.

Strange Usages.

In some instances interesting side-lights are thrown upon usages which to us seem incredible. Thus, for example, he speaks of the urine of the patients in one hospital, probably in a cloth manufacturing town, being sold as a perquisite of the nurses. In another hospital patients were found lying in loose straw upon the floor. In a third (this was in Ireland) the bath house had been converted into a pig pen, and sheltered the pigs belonging to the warden of the hospital. In still another two patients were found lying in the bath tub for the lack of any other bed. He speaks highly of the plans of the hospital at Plymouth, and gives a sketch of the ground plan and elevation, which suggest a modern hospital on the pavilion plan. He also commends the Hotel Dieu at Lyons in France.

### Howard's Ideas of a Good Hospital.

As a result of his observations and investigations he presents the following summary of what he regards as the *desiderata* of a good hospital, which I will present in his own words:

The situation of an infirmary or hospital should be on elevated ground, near a stream of water, and out of a town. The wards, if only one for each sex, to be from twenty-five to thirty feet high, arched, and without apartments over them; otherwise, the building to consist of only two stories, besides the cellars, and the area extended as far as necessary upon this plan, that the inconvenience of higher rooms may be avoided. The first floor raised four or five steps from the ground and the ascent made easy to the entrances. The wards 15 feet high to the ceilings, and distinct ones for medical and chirurgical patients. Two doors to each ward, one of them iron or latticed or canvas. Staircase of stone, spacious, convenient, and easy, as in Italy, Marseilles Malta, etc. No room to contain more than eight beds. The windows lofty and opposite, or circular apertures (as at Leeds Infirmary) opening into passages not less than 6 feet wide; hasps and staples to the upper sashes to prevent their being shut at improper times, or like those at Guy's Hospital. A stone gallery for more readily opening and shutting the windows, as in the Italian hospitals. The ceilings lathed and plastered and proper apertures in them. The fireplaces in the middle of the longer side of the wards. The beds in spacious recesses, as at Toledo and Burgos; or to each bed a recess with curtains, as at Genoa, Savona, etc. The bedsteads iron, painted, and with a screw, that the backs may be easily raised or lowered. The beds on varnished boards or laths, with hair mattresses. In each ward a cistern, basin, and towel for the patients. Vaults on the outside of the wards, and water-closets as at Guy's Hospital, For every improvement that may render such places less offensive should be carefully adopted in all houses containing a number of inhabitants. Airy rooms and refectories for convalescent patients, one spare and unfurnished ward; each ward to be taken in succession and called the spare ward. The kitchen, washhouse, brewhouse, and bakehouse out of the house; but if the kitchen be in the house it should be lofty, as in Christ's Hospital (not underground) and the entrance through the servants' hall. A convenient bath with an easy descent into it. A piazza and spacious walk to induce patients to take the air and exercise. The wards washed once a week—scraped and lime-whited at least once a year. (The machines at Northwich for supplying the salt mines with fresh air, being on a simple construction, would be of admirable use in hospitals, especially if situated in close and confined places.) The patients washed at their admission in the cold or warm bath, and to conform strictly to the rules of nicety and cleanliness.

### General Conclusions.

These are wise words, and would serve as an excellent guide for those persons who are called upon to-day to plan hospitals. It is remarkable that one who had received no special instruction in hygienic matters should have seized so quickly upon the vital points of good hospital construction and management. Everywhere he insists upon fresh air, ventilation, cleanliness, and good food as all-essential to the proper treatment of the sick. No one to-day preaches this gospel more loudly, or with greater clearness, than did this John the Baptist of hospital reform more than a century ago.

In closing this brief paper, I am moved to speak of two facts as suggested by the interesting volumes under consideration. First, the interdependence of all forms of philanthropic work. Howard, in the line of his duties as High Sheriff of Bedfordshire, had his attention called to abuses which existed in the gaols of England, and in his efforts to correct these evils he became interested in reforms in prisons and bridewells. He saw need for similar reforms in almshouses, hospitals, orphan asylums, lazarettos, and receptacles for military prisoners. He became interested in gaol fever, small-pox, plague, and other contagious diseases. There was not a branch of prison or charitable work which was not energised by his devotion and self-sacrificing labours. Second, the work accomplished by a comparatively mediocre man. He was not a scholar or possessed of high attainments in any branch of science. He had simply the ability to investigate thoroughly and to observe closely. The array of impregnable facts which he collected by painful and laborious travels throughout Europe enabled him to initiate reforms far-reaching, and well calculated to bless the race for generations to come.

